# Effects of clonal fragmentation on *Pyrrosia nuda* depend on growth stages in a rubber plantation

**DOI:** 10.3389/fpls.2024.1371040

**Published:** 2024-04-29

**Authors:** Xiaocheng Yu, Nan Jin, Rong Bai, Yuxuan Mo, Xiaoyan Pu, Jingchao Li, Hua-Zheng Lu

**Affiliations:** ^1^ CAS Key Laboratory of Tropical Forest Ecology, Xishuangbanna Tropical Botanical Garden, Chinese Academy of Sciences, Mengla, China; ^2^ National Forest Ecosystem Research Station at Xishuangbanna, Xishuangbanna Tropical Botanical Garden, Chinese Academy of Sciences, Mengla, China; ^3^ College of Life Sciences, University of Chinese Academy of Sciences, Beijing, China; ^4^ Yunnan Provincial Forest Ecosystem Research Station at Xishuangbanna, Xishuangbanna Tropical Botanical Garden, Chinese Academy of Sciences, Mengla, China; ^5^ Party School of the Xishuangbanna Dai Nationality Autonomous Prefecture Committee of Communist Party of China, Yunnan Xishuangbanna Dai Nationality Autonomous Prefecture Committee and State Government, Jinghong, China

**Keywords:** clonal fragmentation, rubber plantation, physiological integration, restoration potential, epiphytic ferns

## Abstract

**Introduction:**

Clonal fragmentation helps to assess clonal plants' growth resilience to human and environmental disturbance. Although clonal integration in epiphytes in tropical rubber plantations is important to understand their role in enhancing biodiversity and ecosystem services, research on this subject is limited. These plantations are typically monospecific economic forests that face increased anthropogenic disturbances.

**Methods:**

In this study, we selected the clonal fern *Pyrrosia nuda* to study its survival status, biomass, maximum quantum yield of photosystem II (F_v_/F_m_), and frond length in response to the level of clonal fragmentation in a tropical rubber plantation.

**Results and discussion:**

The results showed that (1) clonal fragmentation significantly negatively affected the survival rate, biomass, and frond length of clonal plants, but with minimal effects on F_v_/F_m_ at different growth stages; (2) the performance of a ramet (e.g., biomass or frond length) increased with ramet developmental ages and decreased with the number of ramets in a clonal fragment. The age-dependent impacts of clonal fragmentation provide insights into the biodiversity conservation of epiphytes and forest management in man-made plantations. Therefore, to better conserve the biodiversity in tropical forests, especially in environment-friendly rubber plantations, there is a need to reduce anthropogenic disturbances and alleviate the level of fragmentation.

## 1 Introduction

Land-use change by human activities has brought extensive deforestation and alterations in various land types worldwide. This has not only severely damaged the species composition and biodiversity of tropical rainforests but also led to drastic changes in temperature and precipitation patterns in the tropics (Choat et al., 2012; Polson et al., 2016). These natural and anthropogenic disturbances have impacted the growth, flowering, and reproduction of plants (Willmer, 2012; Zambrano and Salguero-Gomez, 2014; Ruiz et al., 2018). The impacts of disturbances have directly or indirectly limited the availability of biotic resources crucial for human sustenance. To meet human resource demands, tropical rainforests are often exploited for agricultural, livestock, and forestry activities such as timber production. Unfortunately, such activities have undermined the structural and functional sustainability of tropical forests, resulting in insufficient ecological services to meet human needs. This exacerbates deforestation in biodiversity hotspots, destroying habitats and disrupting ecosystem equilibrium, and ultimately leading to the degradation and fragmentation of tropical forests (Jha, 2006; Morton et al., 2013). As a paramount type of monoculture artificial forest arising from land-use changes in tropical forests and as a strategic resource for many tropical nations, rubber plantation possesses indispensable economic and social value. However, because the environmental heterogeneity and microhabitat diversity in rubber plantations are lower compared to tropical rainforests, which makes epiphytes in forest canopies in these artificial plantations are more fragile. They even face the risk of species extinction (Baumbach et al., 2021) due to anthropogenic disturbances and fragmentation (Laurance et al., 2000; Malta et al., 2003; Barlow et al., 2007).

As a crucial refuge for endangered species in the tropics, forest canopies provide heterogeneous and diverse microhabitats for various plants and animals, playing a key role in maintaining flora and fauna diversity, community assembly, and ecosystem stability in tropical forests (Gargallo-Garriga et al., 2021; Roa-Fuentes et al., 2022). Nevertheless, extreme weather and human activities may exert influence on the structure and function (e.g., breaking branches, leaves and treetops, etc.) of the forest canopy, which subsequently reduces individual survival, the spread of species, and biotic interactions of host trees and epiphytes (Barlow et al., 2007; Zellweger et al., 2020). Epiphytes, growing in the tropical canopy for more light and fewer competitors, are essential components of tropical forest flora, contributing to species richness, and structural functionality (Kreft et al., 2004; Ellis, 2012). As epiphytes rely on the supportive structures of their hosts, the habitat fragmentation of tropical forests and clonal fragmentation of clonal epiphytes as a result of natural and anthropogenic disturbances pose direct and indirect threats to the performance and microhabitats of epiphytes. Fortunately, most nonvascular and vascular epiphytes can adapt to canopies with stressful resources and an unstable microenvironment through clonal reproduction (Lu et al., 2016, Lu et al., 2020).

Although the severe impacts of various disturbances on biodiversity and functionality can be mitigated by forest canopies and clonal reproduction, they could still negatively affect the productivity and lifespan of sensitive epiphytes, leading to cascading effects (Foster, 2001; Nadkarni and Solano, 2002; De Frenne et al., 2019). Though one of the most natures of clonal plants is physiological integration, which means sharing resources and information within interconnected fragments of a clone (Suzuki and Stuefer, 1999; Brezina et al., 2006). However, in previous studies, the whole clonal epiphytes decreased the growth and performance, while they relied on resource sharing within a clone and interspecific facilitation within canopy communities to mitigate severe stresses and negative effects (Lu et al., 2016, Lu et al., 2020; Dai et al., 2023). In particular, the growth and performance of two clonal plants, *Pyrrosia nummulariifolia* and *Lemmaphyllum microphyllum*, have been found to decline when they rely on clonal integration to deal with the negative effects of clonal fragmentation in a natural limestone forest (Dai et al., 2023). However, our understanding of the impact of clonal fragmentation resulting from extreme weather and human activities on epiphytes in artificial forests and/or plantations remains limited.

With land use changes and human activities, large areas of tropical forests have changed to tropical artificial plantations for decades. Rubber plantations are one of the main man-made forests in the tropics, especially in southeast Asia. The area of rubber plantation is about 573,333 ha in Xishuangbanna, southwest China (Liu et al., 2023; Qi et al., 2023). To improve the quality and efficiency of rubber plantations, researchers have focused on how to structure an environment friendly rubber plantation with higher biodiversity and interspecific facilitation to balance economic efficiency and ecological efficiency. In fact, epiphytes in artificial plantations play an important role in biodiversity maintenance in the tropics (Zheng et al., 2015; Bohnert et al., 2016). However, in rubber plantations, in addition to the disturbance and damage of clonal epiphytes by extreme weather and animals, rubber tapping will cut the clonal organs of epiphytes into several parts on rubber trees, and general management may also destroy and clear the whole cluster of epiphytes. The frequent occurrences of clonal fragmentation in clonal plants can lead to the splitting of intact clonal plants into potentially clonal individuals connected by one or a few ramets (Song et al., 2013b). Different degrees and/or levels of clonal fragmentation could be a function of the survival rate and individual performance of epiphytes in artificial plantations. For understanding the ecological strategies of clonal plants and maintaining biodiversity in man-made forests, it is crucial to study how clonal epiphytes respond to fragmentation in artificial plantations with various disturbances. It also holds practical significance for managing fragmented artificial forests and mitigating habitat disruption caused by human activities (Dong et al., 2012; Wang et al., 2018).

Clonal integration plays a crucial ecological role in mitigating the effects of clonal fragmentation caused by nature and humans. To improve the health of plant systems and the conservation of canopy biodiversity, it may be pertinent to reduce the level of fragmentation and promote system connectivity (Zotz and Hietz, 2001; Grimshaw et al., 2005; Zhang et al., 2009). Numerous studies have found that native plants and invasive plants in both wetland and terrestrial habitats exhibit excellent clonal growth capabilities under clonal fragmentation with severe disturbances, allowing rapid growth and reproduction not for native species but for invasive plants (Dong et al., 2012; Li et al., 2013; Zhang et al., 2023). It means that the physiological integration of resources could be varied in distances or extents for different native and invasive species. Thus, clonal fragments with different numbers of ramets (levels of fragmentation) of various plants in varied forests (e.g., natural and artificial forests) will respond to disturbances differently. Furthermore, various plant species or plants with different developmental stages may rely on clonal integration to different degrees and in terms of resource sharing and resilience of diseases. In general, younger stage individuals depend on resource sharing much more for survival and growth than older ones, for the distinction of resource absorption capability and resilience of stresses. However, little is known about the dependence degree of physiological integration on developmental ages for clonal epiphytes in artificial plantations.

To investigate the impact of clonal fragmentation (i.e. the extents of physiological integration) and its dependence on the development ages of a clonal epiphyte in an artificial forest, this study was carried out as a field in-situ experiment of *P. nuda* in the rubber plantation in Xishuangbanna Tropical Botanical Garden, Chinese Academy of Sciences. This study aims to address the following research questions: 1) Does increasing levels of clonal fragmentation correspond to greater negative impacts on *P. nuda*? 2) What is the effect of developmental stage on the survival and performance of *P. nuda*? 3) Do the effects of clonal fragmentation depend on the relative age of *P. nuda* in rubber plantation?

## 2 Materials and methods

### 2.1 Study site

The study site is located in Xishuangbanna Tropical Botanical Garden (XTBG) (21°55′39″N, 101°15′55″E, 580 m a.s.l.) in MengLun, Xishuangbanna Prefecture, Yunnan Province, China. As a biodiverse hotspot, the topography of Xishuangbanna features mountains and-valleys, with the Hengduan Mountains running north-south, covering approximately 95% of the region’s covered by mountains and hills. This region has a typical tropical monsoon climate and is characterized by a half-year dry season (November-April) and a half-year rainy season (May-October) (Dai et al., 2023). According to the observation data from Xishuangbanna Station for Tropical Rainforest Ecosystem Studies (XSTRES), the monthly mean air temperature is approximately 22.5°C, and the mean annual precipitation is approximately 1500 mm (from 2005 to 2018), with more than 80% occurring during the rainy season. The soil in the study site is Ferralic Cambisol with a pH of around 5.0 developed from sandstone-derived alluvial deposits, which has a clayey texture with 23% coarse sand (2.0 - 0.05 mm), 30% silt (0.05 - 0.002 mm), and 47% clay (< 0.002 mm) and (Yang et al., 2020). The rubber plantation is a predominantly monoculture artificial community, and the rubber trees were cultivated at a spacing of 2.0 m × 4.5 m along the terraced slope following the clearance of primary vegetation in the late 1980s, and the most dominant epiphytic fern species is *P. nuda* in Xishuangbanna.

### 2.2 Experimental design

We selected *Pyrrosia nuda*, the most dominant clonal epiphytic fern, as the target species for *in situ* field experiments in the canopy of a rubber plantation. *P. nuda* has long creeping rhizomes of 1.2-2.1 mm in diameter and in cross section usually with a single, central sclerenchyma strand (State Key Laboratory of Systematic and Evolutionary Botany, 2023). Most *Pyrrosia* species are drought-tolerant (Wei et al., 2017; Derzhavina, 2020).

**Figure 1:**
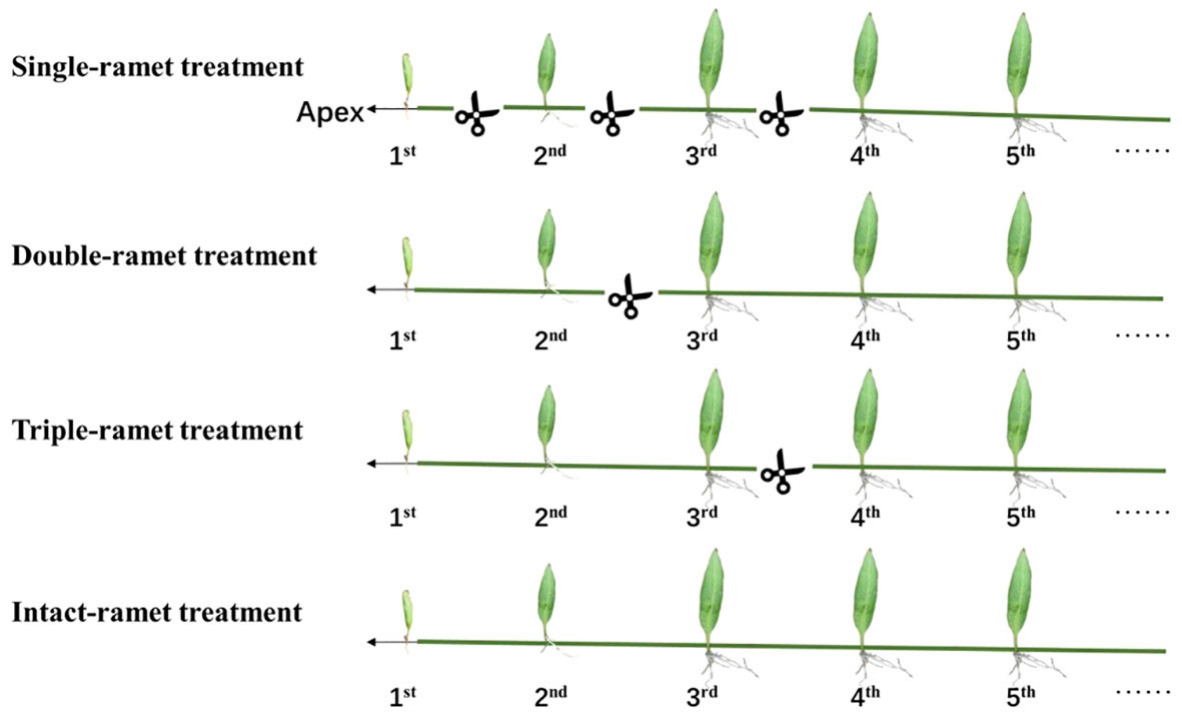
Experimental design. For each of the three epiphytic ferns and each of the 20 host trees, we selected four rhizomes with interconnected ramets. Within each rhizome, we identified the three adjacent ramets closest to the rhizome apex (labeled as 1^st^, 2^nd^, and 3^rd^). In the single-ramet treatment, we severed the rhizome midway between any two adjacent ramets among the first four ramets closest to the rhizome apex. For the double-ramet treatment, we severed the rhizome midway between the 2^nd^ and 3^rd^ ramet closest to the rhizome apex. In the triple-ramet treatment, the rhizome was severed midway between the 3^rd^ and the 4^th^ ramet closest to the rhizome apex. The intact-ramet treatment involved leaving the rhizome intact.

The experiment involved ramets at three developmental stages/ages (1^st^, 2^nd^, and 3^rd^ ramet closest to the rhizome apex) with fiddlehead frond ramets, just-extended frond ramets, and mature frond ramets and included four levels of fragmentation as the treatment—single-, double-, triple- and intact-ramet treatments (see **Figure 1**). Based on previous experiments on clonal integration of epiphytic ferns, it is noted that water stress during the dry season may lead to the death of most single ramets; therefore, this study was focused on the influence of clonal fragmentation on the survival and performance in the rainy season with less water stress (Lu et al., 2015; Zhang et al., 2019; Lu et al., 2020; Zhang et al., 2020b; Dai et al., 2023).

At the beginning of the rainy season, we selected 20 host trees with similar diameter at breast height (DBH) and tree height, which had *P. nuda* as the dominant epiphyte species in the rubber plantation. These rubber trees were planted at equal intervals, and the epiphytes were located in the same canopy height with consistent light conditions and humidity (Liu et al., 2018, 2020; Zeng et al., 2018; Lu et al., 2020). First, we selected a clone of interconnected ramets, then we chose four rhizomes. For each rhizome, we chose the three youngest ramets closest to the rhizome apex as our target materials (labeled as the 1^st^, 2^nd^, and 3^rd^ ramet). We conducted in-situ experiments on epiphytic ferns on each host rubber tree at the height of 2 to 4 m on the trunk below the first branches, with 20 replications for each treatment as below. In the single-ramet treatment, we severed the rhizome midway between any two adjacent ramets of the first four ramets closest to the rhizome apex so that each of the three target ramets (1^st^, 2^nd^, and 3^rd^) was isolated from the rest of the clone. In the double-ramet treatment, we severed the rhizome midway between the 2^nd^ and the 3^rd^ ramet closest to the rhizome apex so that the first two ramets were still connected but isolated from the rest of the clone. In the triple-ramet treatment, we severed the rhizome midway between the 3^rd^ and the 4^th^ ramet closest to the rhizome apex so that the three target ramets were still connected but isolated from the rest of the clone. In the intact-ramet treatment, we left the rhizome intact so that the three target ramets remained connected and connected to the rest of the clone (**Figure 1**).

After 150 days, we assessed the survival status of each of the three target ramets (1^st^, 2^nd^, and 3^rd^ ramet) of *P. nuda*, and harvested each survived ramet *in situ* experiment after measuring its frond length. A ramet was considered dead once all of its fronds were shed, dried, or withered. Biomass was determined after drying at 70 ^°C^C for 48 h. Before harvesting, we assessed the maximum quantum yield of photosystem II (F_v_/F_m_) as a measure of photosynthetic capacity in *P. nuda* using a portable fluorometer (FMS-2, Hansatech, UK). The dark adaptation process involved securing healthy leaves with a Dark Leaf Clip (DLC-8) for a minimum of 20 minutes, avoiding the main veins. Subsequently, we measured the initial fluorescence (F_0_) by exposing the leaves to measuring light (< 0.5 μmol·m^-2^·s^-1^), followed by measuring the maximum fluorescence (F_m_) using a saturating pulse (2800 μmol·m^-2^·s^-1^). This process was repeated, and the variable fluorescence (F_v_) was calculated as F_v_ = F_m_ – F_0_. The maximum photochemical quantum yield of photosystem II (F_v_/F_m_) was then determined using the formula F_v_/F_m_ = (F_m_ – F_0_)/F_m_, as described in previous studies (Bolharnordenkampe et al., 1989; Heidbüchel and Hussner, 2020). After allowing fluorescence values to stabilize (approximately three to five minutes), we recorded the actual quantum yield and other fluorescence parameters. This standardized methodology ensures a precise assessment of the photosynthetic capacity in *P. nuda* fronds. F_v_/F_m_ was calculated with the equation F_v_/F_m_ = (F_m_ – F_0_)/F_m_, where F_0_ and F_m_ are the minimum and maximum fluorescence yield of a dark-adapted sample after a saturation pulse (>5000 μmol photons m^−2^s^−1^ of actinic white light), respectively (Bolharnordenkampe et al., 1989; Lu et al., 2020). F_v_/F_m_ reflects the photosynthetic performance and stress resistance of plants (Butler and Kitajima, 1975; Wei et al., 2019; Li et al., 2020), which indirectly indicates the ability of plants to absorb and the strength of plants to utilize light energy for photosynthesis. This value is positively correlated with plants’ growth and regeneration ability. Therefore, higher F_v_/F_m_ can indirectly indicate greater survival rate, biomass, and frond length of epiphytic ferns, serving as an indicator to evaluate the growth ability and status of epiphytes (Gauslaa et al., 2001; Malta et al., 2003).

### 2.3 Data analysis

The data were analyzed using (generalized) linear mixed models with R version 4.2.0 (R Core Team, 2022). The “glmer” function in the “lme4” package and the “lmer” function in the “lmerTest” package were employed (Bates et al., 2015; Kuznetsova et al., 2017). Initially, the fixed factors included fragmentation level (single-, double-, triple- and intact-ramet treatments), developmental stage/age (1^st^, 2^nd^, and 3^rd^ ramet closest to the rhizome apex), and their interactions. For individual survival data with binomial error distributions, a generalized linear mixed model was then applied (“glmer” function), with the level of clonal fragmentation and age as fixed factors, the initial individual height (leaf size) as covariate and the host tree in the sampling site as a random variable. For the biomass, F_v_/F_m_, and frond length of surviving ramets, a linear mixed model was used (“lmer” function), with the level of clonal fragmentation and age as fixed factors, the initial individual height (leaf size) as covariate and the host tree in the sampling site as a random variable. The data were log-transformed if needed before analysis to improve the normality of the residuals.

In the (generalized) linear mixed models, log-likelihood ratio tests were conducted to assess the significance of the fixed factors (Bedogni, 2010). During the post-hoc analyses of significant differences among treatments, a ramet was considered dead and its data was treated as missing when all of its fronds were shed, dried, or withered. In performing the log-likelihood ratio test, the null data were excluded in the analysis. These tests involve comparing a model with the term of interest to a model without the term of interest. The calculated log-likelihood ratios approximated a χ^2^ distribution (Platt et al., 2004). Specifically, we sequentially removed the two-way interactions, each of the two key factors and the covariate, and then compared the fit of the simplified model to the more complex model. The statistical difference in model fit indicates that the effect of the removed factor is significant.

## 3 Results

**Figure 2:**
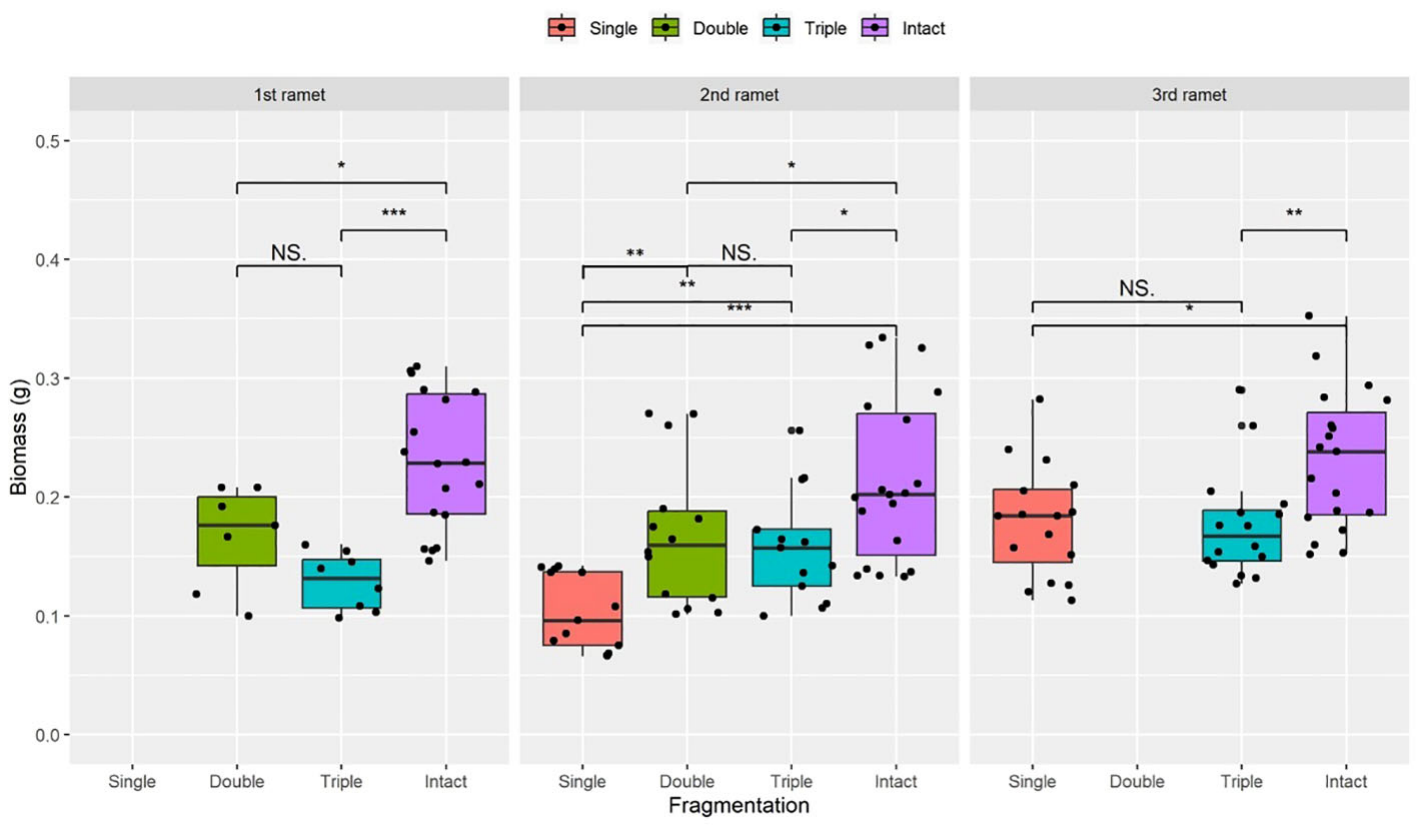
Biomass of surviving clonal ramet at different growth stages of each clonal fragment (single, double, triple, control/intact). Significant differences between each treatment is denoted by asterisks::*P < 0.05; **P <0.01; and ***P < 0.001; NS indicates non-significant differences between each treatment (P  ≥ 0.05); Within each rhizome, the three adjacent ramets closest to the rhizome apex (labeled as 1^st^, 2^nd^, and 3^rd^).

**Figure 3:**
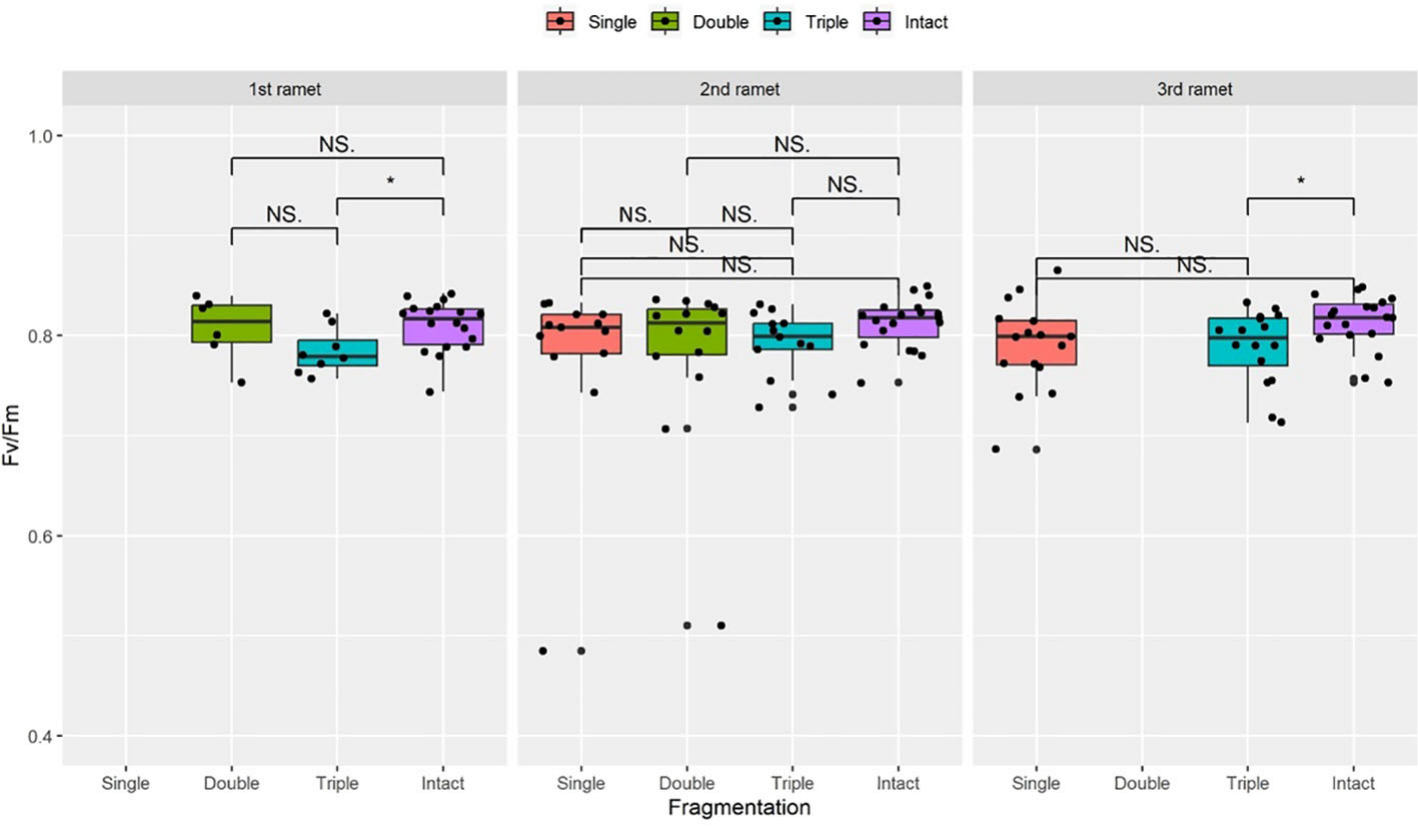
The maximum quantum yield of PS II (F_v_/F_m_) surviving clonal ramet at different growth stages of each clonal fragment (single, double, triple, control/intact). Significant differences between each treatment is denoted by asterisks:*P < 0.05; NS indicates non-significant differences between each treatment (P  ≥ 0.05); Within each rhizome, the three adjacent ramets closest to the rhizome apex (labeled as 1^st^, 2^nd^, and 3^rd^).

**Figure 4:**
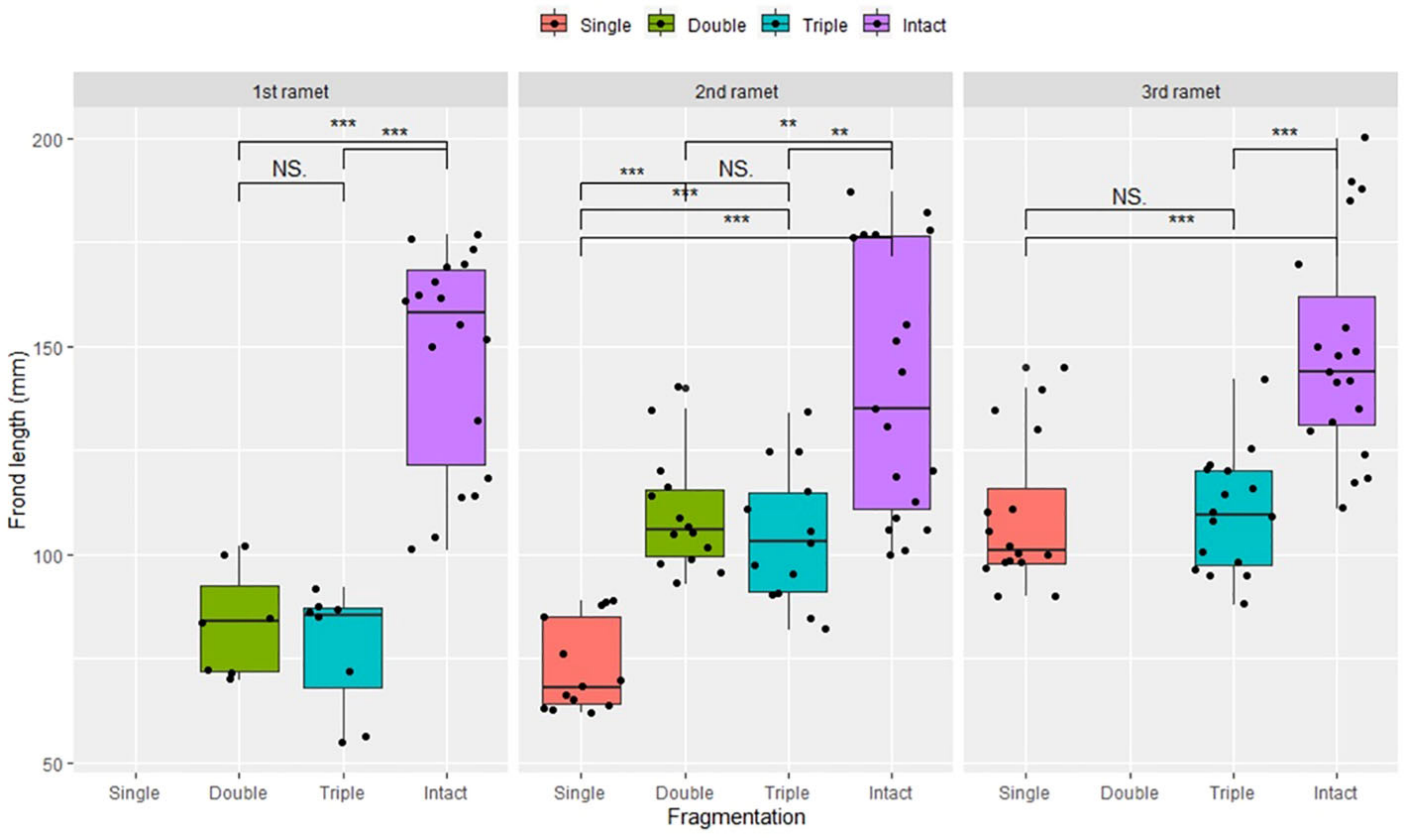
Frond length of surviving clonal ramet at different growth stages of each clonal fragment (single, double, triple, control/intact). Significant differences between each treatment is denoted by asterisks::*P < 0.05; NS indicates non-significant differences between each treatment (P  ≥ 0.05); Within each rhizome, the three adjacent ramets closest to the rhizome apex (labeled as 1^st^, 2^nd^, and 3^rd^).

Compared to the intact-ramet treatment, clonal fragmentation significantly reduced the clonal ramet survival, surviving ramet biomass, F_v_/F_m_, and frond length of *P. nuda*. With the increasing levels of clonal fragmentation, the negative effects of fragmentation increased (**Table 1**, **Figures 2**–**4**). In *P. nuda*, the fragmentation led to a marked decrease in ramet biomass, with single- (0.145 ± 0.010), double- (0.167 ± 0.011), and triple-ramet (0.159 ± 0.007) treatments had lower biomass than the intact-ramet (0.224 ± 0.008). The F_v_/F_m_, although reduced across all fragmentation levels (single- (0.785 ± 0.012), double- (0.785 ± 0.012), triple-ramet (0.789 ± 0.005)) relative to the intact-ramet (0.811 ± 0.003), showed no difference among the three severed treatments. Notably, frond length experienced a sharp decline from the intact-ramet (145.55 ± 3.73) to single- (93.06 ± 4.41), double- (93.06 ± 4.41), and triple-ramet (101.10 ± 3.20) treatments (**Figure 4**).

**Table 1:** Effects of fragmentation level (F), growth stage (S) and their interaction (F × S) on survival, growth, the maximum quantum yield of PS II (F_v_/F_m_), and frond length of *P. nuda* in (generalized) linear mixed models.

Fixed factors	Survival			Biomassζ			F_v_/F_m_ζ			Frond lengthζ		
	df	χ^2^	*p*	df	χ^2^	*p*	df	χ^2^	*p*	df	χ^2^	*p*
Fragmentation (F)	3	50.6	<0.001	3	95.4	<0.001	3	9.2	0.026	3	184.3	<0.001
Stage (S)	2	0.9	0.651	2	62.6	<0.001	2	0.5	0.783	2	49.8	<0.001
F × S	5	15.9	0.007	4	62.0	<0.001	4	3.4	0.487	4	260.3	<0.001
Frond sizeζ	1	49.6	<0.001	1	9.5	0.002	1	0.3	0.600	1	13.3	<0.001

ζdata were log-transformed. Fragmentation level, and growth stage, were treated as fixed effects, host trees nested in study sites as random factors, and original plant height as covariate. “df” means the degrees of freedom, “χ^2^” is the value of the chi-square test, “p” is the significance of the test.

The growth stage significantly influenced biomass and frond length of surviving ramet. Specifically, as ramets aged, both biomass and frond length increased. However, it did not affect the clonal ramet survival and F_v_/F_m_ of surviving ramets of *P. nuda*, indicating higher survival and biomass in plants at the mature leaf stage (**Table 1**, **Figures 2**, **4**). The older the ramet, the better the ramet grew during clonal fragmentation (**Figures 2**, **4**).

There were significant interaction effects of clonal fragmentation and developmental stage on the clonal ramet survival, surviving ramet biomass and frond length of *P. nuda*. However, no such effects were observed for F_v_/F_m_ of surviving ramets (**Table 1**, **Figures 2**-**4**). It meant that the negative influences of clonal fragmentation depend on growth stages, i.e. the survival rate and growth of younger ramets in small fragments (higher fragmentation levels) was lower than that in large fragments (lower fragmentation levels) and the difference was much lower for older ramets.

## 4 Discussion

### 4.1 Response of *P. nuda* to clonal fragmentation

As shown in the results, clonal fragmentation had a significantly negative effect on clonal ramet survival, biomass, F_v_/F_m_ and frond length of surviving ramets of *P. nuda*, especially in the fiddlehead fern stage (P <0.001, **Figures 2**-**4**). This result is consistent with previous findings. There were negative effects of clonal fragmentation on the survival rate and biomass of two clonal ferns *Pyrrosia nummulariifolia* and *Lemmaphyllum microphyllum* with different ecotypes of epiphytic and lithophytic clonal ramets (Dai et al., 2023). This confirmed our initial research hypothesis in that the higher the fragmentation level, the more severe the negative influence. This result suggests that epiphytes in both limestone forests and artificial forests would decrease the survival rate and performance by clonal fragmentation resulting from natural and anthropogenic disturbances. Similar to fern species, the negative affects of clonal fragmentation also decreased the competitive ability and the number of ramets of seed plants. As clonal fragmentation significantly inhibits the growth of *S. natans*, it will result in a notable reduction in its competitive fitness. This underscores the detrimental impact of clonal fragmentation on the overall competitive capacity of the species (Dong et al., 2012; Zhang et al., 2020a). Clonal fragmentation destroys the interconnection spacers and narrows the range of physiological integration, resulting in resource sharing within a limited zone. Clonal epiphytes, such as *Selliguea griffithiana*, rely on physiological integration for surviving and growing in both canopy and understory habitats, especially in the canopies (Lu et al., 2015), so do some invasive or wetland clonal plants (Back et al., 2013; Song et al., 2022). However, clonal fragmentation has been observed to significantly augment the growth potential of certain aquatic and invasive plants, as detailed in research by Zhang et al. (2019), and Rosef et al. (2020). These studies have collectively demonstrated the consequential enhancement of proliferation and regenerative capabilities arising from such fragmentation (to compete with local species and occupy the new habitats). Moreover, specific attention has been directed towards the advantageous effects of root fragmentation in fast-growing and ecologically adaptable species, as highlighted by Huber et al. (2014).

In this study, the epiphytic fern of *P. nuda* could provide more support for survival and growth from interconnected clonal ramets under natural and anthropogenic disturbance. The adverse effects on survival rate and frond length are likely due to the limited resources for growth and adaptation in the juvenile fiddlehead leaf stage of *P. nuda* ramets subjected to fragmentation, leading to worse self-adjustment and adaptability of clonal ramets after disturbance (Rudolphi and Gustafsson, 2011; Lu et al., 2015; Song et al., 2022; Dai et al., 2023), and such effects on biomass are likely to be the inability of ramets to share resources between them, as the reduced regeneration capacity and the increased risk of infection after ramet fragmentation can prevent the allocation and share of resources between ramets (Song et al., 2013b; Lu et al., 2020). As the level of clonal fragmentation decreases, there is a positive effect on ramet survival, biomass, and frond length, which could be because of the connection between ramets allowing epiphytes to obtain more shared resources and to better cope with stresses (Lu et al., 2015, Lu et al., 2020; Dai et al., 2023) and resulting in improved growth and reproductive regeneration capabilities. There is also a significant effect on the clonal ramet F_v_/F_m_, which is likely because the fragmentation process leads to water deficiency in fragmented ramets, resulting in an insufficient supply of essential raw materials for photosynthesis (Wang et al., 2020). Rubber plantations and epiphytes in the canopy are susceptible to weather and disturbances, which, together with water scarcity that reduces carbon dioxide movement into the fronds, can lead to a lack of carbon and energy demand for photochemistry and photosynthesis (Zheng et al., 2015; Wei et al., 2019; Li et al., 2020).

### 4.2 Response of *P. nuda* to different growth ages

There were significant effects of developmental age on biomass and frond growth; that is, the higher the ramet developmental age, the larger the ramet biomass and frond length. Similar findings in previous studies suggest that this might be attributed to the insufficient self-regulation capacity of epiphytes, lack of nutrient reserves and rapid growth capability during the early stages of ramet formation when just-extended frond ramets are prevented from resource sharing (Lu et al., 2015; Lin et al., 2018). For example, the rooting position significantly influenced the growth of individual ramets, with the second and third most apical ramets exhibiting optimal growth when positioned as the most apical rooted ramet. This effect was more pronounced under higher nitrogen levels (Wang et al., 2017). In addition, the rapid development of clonal ramet root systems in the extended frond and mature stages potentially broadens their ecological niche, making them less susceptible to the affects of fragmentation (Truscott et al., 2006). As plant age increases, mature ramets share and transfer nutrients to young ramets, indicating that the growth of young ramets depends more on the closely related mature maternal ramets (Alpert, 1999; de Kroon et al., 2005; Song et al., 2013a). Therefore, severing the connection between the clonal offspring and the older parents’ ramets at a younger age may result in inadequate nutrient supply, and hindered sharing of water resources and photosynthetic products, thereby reducing their survival and growth rate.

The adjustment and adaptive performance of the 1^st^ age younger fragmented clonal ramets may diminish after disturbance, with their reproductive and resistance capabilities enhanced with growth stage and maturity (Rudolphi and Gustafsson, 2011). There is relatively little variation in the impact on F_v_/F_m_ and ramet survival with increased ramet developmental age (**Table 1**, **Figures 2**-**4**), which is in contrast to previous studies which, indicated that clonal integration has varying effects on the maximum photosynthetic efficiency of ramets. Clonal integration significantly influences the photosynthetic rates of *Alternanthera philoxeroides* and *Fragaria vesca* but does not affect the photosynthetic rate of the epiphytic fern species *Diplopterygium glaucoma* (Luo et al., 2014; Reynolds et al., 2014). The yield effect of benefits and resource acquisition does not necessarily rely entirely on the frond photochemistry to obtain the energy required for growth. The lack of significance could be linked to the depletion of most plant resources and energy for recovering damage after fragmentation. The impact on leaf photosynthesis can translate into varying effects on ramet survival and growth. Therefore, an extended period of resource accumulation is required to enhance plant photosynthetic performance and stress resistance (Wei et al., 2019; Li et al., 2020; Lu et al., 2020). Furthermore, the presence of the forest canopy may block some light, resulting in insignificant effects on F_v_/F_m_ at different growth stages for clonal ramets situated in the canopy, under normal circumstances, the F_v_/F_m_ in plants typically ranges from 0.70 to 0.80 (Butler and Kitajima, 1975; Bolharnordenkampe et al., 1989). After disconnection, the F_v_/F_m_ is significantly reduced. The changes in the F_v_/F_m_ depend on the species and the light condition of the plants (Wei et al., 2019; Li et al., 2020). The negative impact of clonal fragmentation on epiphytes is age-dependent and influenced by clonal integration effects (Lu et al., 2016, Lu et al., 2020).

### 4.3 High dependence of clonal fragmentation on growth stage

The interaction between the level of clonal fragmentation and the developmental stage of the ramet showed that clonal fragmentation had entirely different effects on the survival, biomass, and frond length of *P. nuda* ramets at different growth ages. This is consistent with previous research which found *Pyrrosia nummulariifolia* and *Lemmaphyllum* microphyllum exhibit significant interaction effects of clonal fragmentation and ages on ramet survival and growth in natural forests (Dai et al., 2023). This appears to stem from the transient reliance of relatively early-maturing clonal ramets on clonal integration (Saine et al., 2018). Slow-developing or mature epiphytes may exhibit a higher dependence on growth stages. The impact of clonal fragmentation on clonal ramet growth and dependence on clonal integration varied with growth stages for the same species, diminishing growth in younger ramets while enhancing growth in older ramets (Stuefer et al., 2001; Dong et al., 2012; Song et al., 2013b; Luo et al., 2014; Wang et al., 2014). Additionally, this age-dependent response to clonal fragmentation might be associated with the clonal division of labor in plants during ontogenesis. Clonal plants undergo morphological or functional specialization at different growth stages, enhancing their ability to acquire heterogeneous resources, thus elevating their position and role in competitive relationships (Fan et al., 2018). Consequently, the capacity of epiphytes to acquire resources varies at different growth stages, leading to distinct affects on clonal ramet survival and growth after clonal fragmentation.

The effect of clonal fragmentation on clonal ramets depended on growth stages. Clonal fragmentation affects early juvenile fiddlehead, extended frond, and mature *P. nuda* ramets differently, with a significantly negative impact on survival during the early growth stage. But there is no significant effect on survival during the extended frond and mature stages. Clonal fragmentation is likely to have a negative impact on the growth of young ramets, while positively influence the growth of older ramets (Ortego et al., 2002; Dong et al., 2012; Zhang et al., 2023). Stored carbohydrates in clonal ramets might be used to prevent the risk of future ramet death (Suzuki and Stuefer, 1999), which could be a result of a ramet response to the current growth potential and a trade-off between the current growth potential and future risk regulation that could explain the observed differences. This is further supported by the enhanced resistance of mature ramets to clonal fragmentation, especially in epiphytic ferns (Wei et al., 2019; Zhang et al., 2023). This highlights the high dependence of clonal integration on growth ages, and resource sharing facilitated by clonal integration contributes significantly to the survival and performance of epiphytic ferns.

## 5 Conclusion

In artificial rubber plantations, clonal fragmentation exerted negative age-dependent effects on survival and performance of the epiphytic fern *P. nuda*, especially at the juvenile fiddlehead leaf stage. When clonal ramets are fragmented by natural or anthropogenic disturbance, the plant’s resource sharing will be limited to fragments of small numbers of interconnected ramets, leading to resource scarcity and declined performance. Such negative effects of clonal fragmentation were much more severe on juvenile individuals than aged ones, which may have resulted from the higher stress-resilience and resource-storage of adults. Based on the results of the survival, biomass, and frond length analysis of the *P. nuda* clonal ramets, it is imperative to minimize and avoid anthropogenic disturbance causing fragmentation in artificial forests, such as tropical rubber plantations, to mitigate its impact on plant biodiversity. This research indicates that clonal integration plays a crucial role in the growth, reproduction, and performance of clonal plants in tropical artificial forests under adverse stress conditions. It sheds light on the biodiversity conservation and maintenance during the establishment of environment-friendly rubber plantations establishment with increased human activities, land-use changes and extreme weather.

## Data availability statement

The original contributions presented in the study are included in the article/supplementary material. Further inquiries can be directed to the corresponding author.

## Author contributions

XY: Data curation, Visualization, Writing – original draft, Writing – review & editing. NJ: Data curation, Supervision, Writing – review & editing, Writing – original draft. RB: Data curation, Writing – review & editing, Visualization, Writing – original draft. YM: Data curation, Writing – review & editing, Visualization, Investigation. XP: Data curation, Writing – review & editing, Investigation. JL: Writing – review & editing, Visualization. H-ZL: Supervision, Writing – review & editing, Visualization, Data curation, Investigation, Writing – original draft, Conceptualization, Funding acquisition, Methodology.
